# Role of berberine in ameliorating *Schistosoma mansoni*-induced hepatic injury in mice

**DOI:** 10.1186/0717-6287-47-8

**Published:** 2014-04-01

**Authors:** Mohamed A Dkhil

**Affiliations:** Department of Zoology and Entomology, Faculty of Science, Helwan University, Cairo, Egypt; Department of Zoology, College of Science, King Saud University, P.O. Box: 2455, Riyadh, 11451 Saudi Arabia

**Keywords:** *Schistosoma mansoni*, Berberine, Liver, Mice

## Abstract

**Background:**

Schistosomiasis is caused by helminth parasites of the genus *Schistosoma*. Berberine chloride (BER), an isoquinoline alkaloid, has been used *in vivo* for its antiparasitic, antioxidant and hepatoprotective properties. In this study, the protective effect of BER and praziquantel has been compared for the extent of schistosomiasis-induced oxidative stress in hepatic tissue of mice.

**Results:**

*S. mansoni* was able to induce inflammation and injury to the liver, evidenced (i) by an increase in inflammatory cellular infiltrations, dilated sinusoids and vacuolated hepatocytes, (ii) by decreased levels of alanine and aspartate aminotransferases and increased levels of alkaline phosphatase, γ-glutamyl transferase in the liver homogenate, (iii) by increased production of nitric oxide and thiobarbituric acid reactive substances, and (iv) by lowered glutathione levels and decreased activities of catalase and superoxide dismutase, respectively. All these infection-induced parameters were significantly altered during BER treatment. In particular, berberine counteracted the *S. mansoni*-induced loss of glutathione and the activities of catalase and superoxide dismutase.

**Conclusion:**

Based on these results, it is concluded that berberine could ameliorate pre-existing liver damage and oxidative stress conditions due to schistosomiasis.

## Background

Schistosomiasis is a chronic, parasitic disease caused by blood flukes (trematode worms) of the genus *Schistosoma*. More than 207 million people are infected worldwide, with an estimated 700 million people at risk in 74 endemic countries (WHO, [1]). Experimental infection of laboratory animals with *Schistosoma mansoni* has been frequently used as a model for the analysis of the pathological and physiopathological aspects of human infection [2].

There is as yet no vaccine available for Schistosomiasis and the current mainstay of control is chemotherapy with praziquantel (PZQ). In view of concern about the development of tolerance and/or resistance to PZQ, there is a need for research into and development of novel drugs for the prevention and cure of schistosomiasis [3]. Moreover, PZQ is associated with considerable adverse clinical effects, some occurring within 24 hours [3]. Although the precise mechanism of action of PZQ has not been clarified, it appears to cause severe spasms and paralysis of the worms’ muscles. This paralysis is accompanied–and probably caused–by a rapid influx of Ca ^2+^ inside the schistosome [4, 5].

Although chemotherapy is still one of the most effective methods for controlling schistosomiasis [6], the value of many of the plant species that have been used throughout the world in traditional medicine for the treatment of both veterinary and human helminthes is increasingly being recognised [7]. Few plants, however, have been screened for activity against adult *Schistosoma sp*., although some medically important species, such as *Zingiber officinale*, *Nigella sativa* and *Asparagus officinalis*, do show an effect against schistosomiasis [8, 9].

Berberine is an isoquinoline alkaloid with a long history of medicinal usage. It is a well-known naturally occurring medicine derived from the root and the stem bark of numerous clinically important medicinal plants such as *Hydrastis canadensis* (goldenseal), *Berberis aquifolium* (Oregone grape), *Berberis aristata* (tree turmeric), *Berberis vulgaris* (barbery), and many other plants [10]. Berberine extracts and decoctions have demonstrated significant antimicrobial and antiparasitic activity against a variety of organisms including bacteria, viruses, fungi, protozoans, helminths and Chlamydia [11]. Extensive research within the past decade indicates that berberine is associated with a wide range of pharmacological effects, including antioxidative [12], anti-inflammatory [13], and immunoregulative [14] activities. Several studies have also demonstrated the inhibitory effects of berberine on chemically induced cytotoxicity, lipid peroxidation and oxidative stress in the liver [15, 16]. In this context, the current study aimed to investigate the role of berberine against schistosomiasis-induced hepatic damage in mice.

## Results

**Figure 1:**
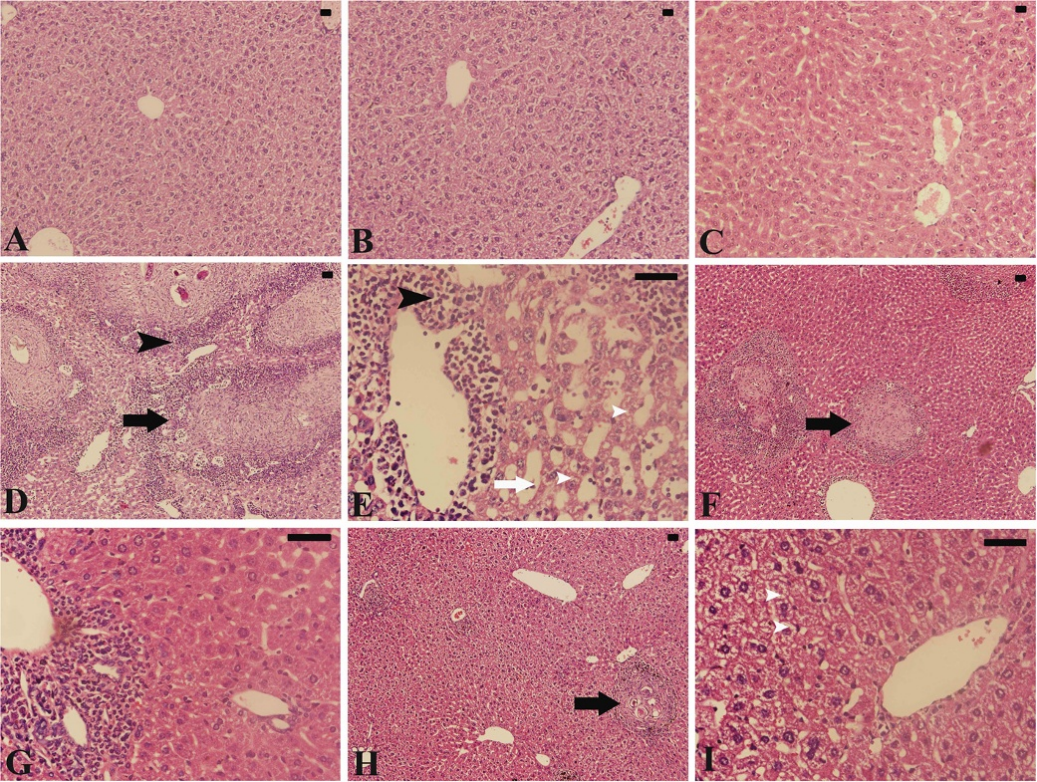
**Histological changes in hepatic tissue of non-infected and infected mice with** ***S. mansoni*** **on day 55 p.i. (A)** Non-infected liver with normal architecture. **(B)** Non-infected, PQZ treated mouse liver with normal structure. **(C)** Non-infected, berberine treated mouse liver with normal structure. **(D,E)** Infected liver from group 4 with prominent inflammation (black arrow heads) especially around granuloma (black arrow), sinusoidal dilatations (white arrow) and hepatocytic vacuolation. **(F,G)** Infected PQZ treated mouse liver from group 5 showing fewer lesions. **(H,I)** Infected berberine treated mouse liver from group 6 showing reduced tissue damage. Sections are stained with hematoxylin and eosin. Bar = 50 μm.

Histological investigation of hepatic tissue sections revealed that *S. mansoni* caused a severe granulomatous inflammatory response in the liver, as indicated by inflammatory cellular infiltration as well as cytoplasmic vacuolation and degeneration of hepatocytes. Granulomas were marked by concentric fibrosis with many fibroblasts encircling the trapped eggs. They were surrounded by a cuff of aggregated lymphocytes, epitheloid cells, eosinophils and collagenous fibres. The presence of numerous granulomas resulted in disorganization of the hepatic strands and lobular structure. In addition, the hepatic sinusoids were dilated and apparently contained more Kupffer cells (Figure 1).

**Figure 2:**
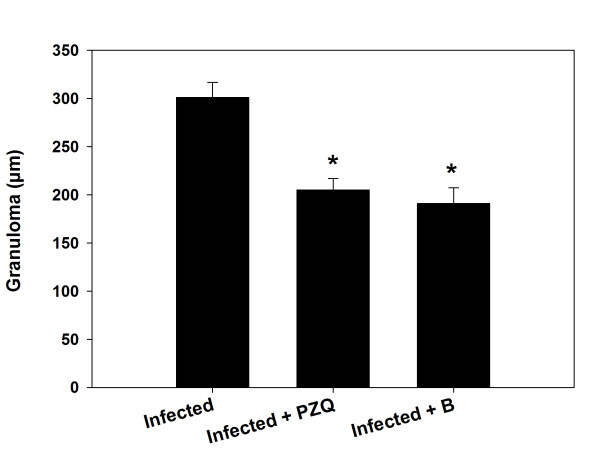
**Reduction in granuloma size in the livers of mice infected with** ***S. mansoni*** **and treated with berberine.** Values are means ± SD (n = 8). *Significant change at *P* ≤ 0.05.

**Figure 3:**
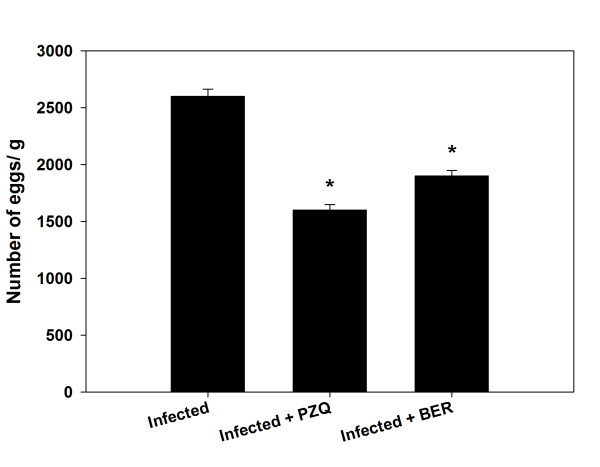
**Egg density in the hepatic tissues of mice infected with** ***S. mansoni*** **and treated with PQZ or berberine.** Values are means ± SD (n = 8). *Significant change at *P* ≤ 0.05.

Berberine administration induced a significant reduction in the size of this granulomatous inflammation compared to the livers of infected untreated animals (Figure 1 and Figure 2). While the diameter of the granulomas reached approximately 300 ± 15.8 μm in infected livers, treatment with berberine significantly reduced their diameter to 190 ± 16.3 μm. This reduction in granuloma size was similar to that in the PZQ treated group (Group 5) (Figure 1 and Figure 2). In the berberine treated group, meanwhile (group VI), the hepatic lobular architecture appeared to be restored its normal organization and most hepatocytes showed a normal appearance, even in the vicinity of the granulomas. Some sinusoids remained dilated and infiltrated with lymphocytes, however, and some Kupffer cells were still hypertrophied (Figure 1). All these alterations are considered through the histological liver activity index compiled according to Ishak et al. [17], which were categorized from 9-16 for the infected livers in comparison to 1-3 for non-infected controls (Table 1). Moreover, mice treated with PQZ and berberine displayed a highly significant increase in the percentage of egg reduction in the liver, estimated as approximately 39% and 27% respectively (Figure 3).

**Table 1:** **Histopathological changes in hepatic tissue of mice infected with** ***S. mansoni***

Group		Microscopic observation					
	Histological activity index ^a^	Necrosis or apoptosis	Hemorrhage	Disorganized sinusoids	Infiltration of lymphocytes	Hyperplasia of Kupffer cells	Hepatocytic swelling
Control	1	0	0	0	0	0	0
PZQ	3	0	0	+	+	0	0
BER	2	+	0	0	+	0	0
Infected	14-16	+++	+++	++	+++	+++	++
Infected + PZQ	9-11	++	+	+	++	++	+
Infected + BER	9-12	+	+	+	++	+	+

^a^Modified according to Ishak et al. [17]. Score: 1–3, minimal; 4–8, mild; 9–12, moderate; 13–18, severe. 0: absent; +: mild; ++; moderate; and +++: severe.

Most previous studies have determined the activities of the liver enzymes in the blood serum. In this study, the activities of ALT, AST, ALP and γ-GT have been measured in the liver homogenates. The activities of ALT and AST in our study were significantly decreased in the liver homogenate of the infected mice (group 3) by approximately 40% and 50%, (*P* < 0.05), respectively (Table 2). Liver ALP and γGT in group 3, meanwhile, were significantly elevated by 55% and 48%, (*P* < 0.05), respectively. Again, both PQZ and berberine treatments were associated with significant changes in these liver parameters, as can be seen from Table 2.

**Table 2:** **Effect of berberine chloride on hepatic ALT, AST, ALP and γGT in male mice infected with** ***S. mansoni***

Experimental groups	ALT	AST	ALP	γ-GT
	(U/g)	(U/g)	(U/g)	(U/g)
Control	304 ± 1.8	229 ± 1.8	16 ± 1	25 ± 0.8
PZQ	315 ± 1.7^a^	220 ± 4.5^a^	13 ± 0.3^a^	18 ± 1^a^
BER	318 ± 1.4^a^	255 ± 2.3^a^	7.4 ± 0.4^a^	15 ± 0.8^a^
Infected	218 ± 3.6^a^	125 ± 1^a^	36 ± 1.2^a^	48 ± 1.6^a^
Infected + PZQ	263 ± 1.3^abc^	146 ± 1.9 ^abc^	17 ± 1^bc^	16 ± 0.5^ac^
Infected + BER	280 ± 2.8^abc^	182 ± 2.4^abc^	12 ± 1.6^abc^	20 ± 0.6^abc^

Values are means ± SD (n = 8). ^a^Significant difference as compared with Group I (*p* ≤ 0.05). ^b^Significant difference as compared with corresponding control group (*p* ≤ 0.05). ^c^Significant difference as compared with infected group (Group IV) (*p* ≤ 0.05).

**Figure 4:**
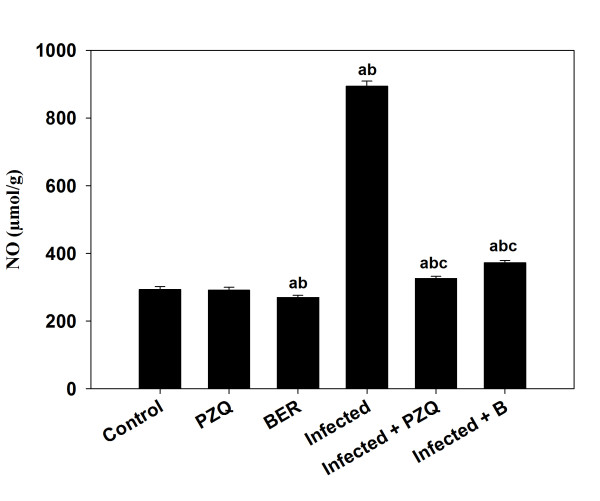
**Effect of berberine on the level of NO in the liver homogenates of mice infected with** ***S. mansoni.***Values are means ± SD (n = 8). ^a^Significant difference compared to Group 1 (*p* ≤ 0.05). ^b^Significant difference compared to the corresponding control group (*p* ≤ 0.05). ^c^Significant difference compared to the infected group (Group 6) (*p* ≤ 0.05).

**Figure 5:**
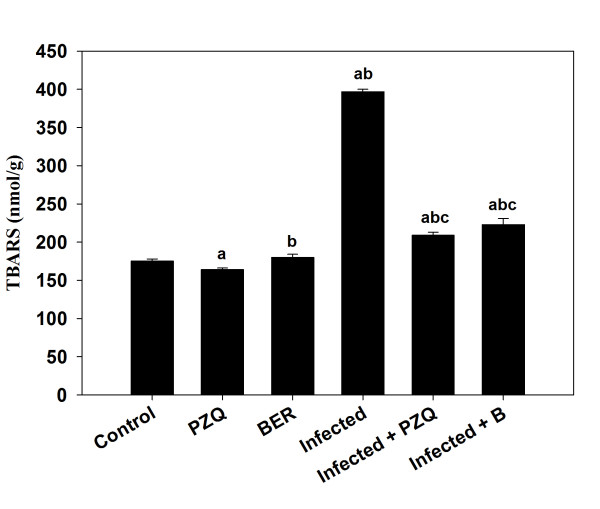
**Effect of berberine on the level of malondialdehyde in liver homogenates of mice infected with** ***S. mansoni.***Values are means ± SD (n = 8). ^a^Significant difference compared to Group 1 (*p* ≤ 0.05). ^b^Significant difference compared to the corresponding control group (*p* ≤ 0.05). ^c^Significant difference compared to the infected group (Group 6) (*p* ≤ 0.05).

*S. mansoni* also induced a highly significant increase in hepatic NO (Figure 4) and MDA (Figure 5) by approximately 3.5 and 2.3 fold, respectively. Treatment with berberine, however, significantly reduced the *S. mansoni*-induced increase in TBARS and NO (Figure 4 and Figure 5). Finally, some major components involved in the down-regulation of substances formed during oxidative stress, such as GSH, catalase and SOD, have been determined (Table 3). It was striking that these substances were significantly down-regulated by *S. mansoni* infection but that these effects were largely prevented by berberine treatment (Table 3).

**Table 3:** **Effect of berberine on liver glutathione, catalase and superoxide dismutase in male mice infected with** ***S. mansoni***

Experimental groups	GSH	CAT	SOD
	(mmol/g liver)	(U/g liver)	(U/g liver)
Control	0.8 ± 0.02	1.3 ± 0.04	1455 ± 8.5
PZQ	1.1 ± 0.02^a^	1.4 ± 0.04	1371 ± 1.4^a^
BER	1.2 ± 0.02^a^	1.6 ± 0.01^a^	1589 ± 6.2^ab^
Infected	0.61 ± 0.02^a^	0.75 ± 0.02^a^	859 ± 1.3^a^
Infected + PZQ	0.76 ± 0.08^ab^	1.3 ± 0.01^bc^	902 ± 2.2^abc^
Infected + BER	0.67 ± 0.02^ab^	1.2 ± 0.03^bc^	924 ± 1.7^abc^

Values are means ± SD (n = 8). ^a^Significant difference as compared with Group I (*p* ≤ 0.05). ^b^Significant difference as compared with corresponding control group (*p* ≤ 0.05). ^c^Significant difference as compared with infected group (Group IV) (*p* ≤ 0.05).

## Discussion

The pharmacologic actions of berberine include metabolic inhibition of certain organisms, inhibition of enterotoxin formation, inhibition of intestinal fluid accumulation and ion secretion, inhibition of smooth muscle contraction, reduction of inflammation and inhibition of platelet aggregation [11]. Praziquantel, meanwhile, works by causing severe spasms and paralysis of the worms’ muscles. This paralysis is accompanied–and probably caused–by a rapid influx of Ca ^2+^ inside the schistosome (Doenhoff et al., 2008). Berberine is not considered toxic at doses used in clinical situations, nor has it been shown to be cytotoxic or mutagenic [11].

In this study, the administration of berberine for 10 days was shown to have a pronounced antischistosomal effect. As Cheever [18] observed, hepatic fibrosis in infected mice is related to egg numbers, i.e. mice with heavier infection have more total hepatic fibrosis. The study showed the relationship between trapped eggs and encircling fibroblasts, supporting the work of Chesney et al., [19], who described the infiltration of circulating fibroblasts into granulomas and speculated that these cells may be important for attracting lymphocytes as well as forming collagen. Our results show the action of berberine in the form of a significant reduction in egg numbers (Figure 3) together with a significant reduction in the size of granulomas, and a decrease in the number of involutive granulomas compared with the infected control mice. Taken together, these findings suggest a possible antifibrotic role of berberine [20, 21]. The commonly encountered hepatic alterations in untreated animals were periportal inflammation, sinusoidal infiltration and Kupffer cell activation these features were reduced in animals treated with berberine, further supporting the suggestion of its fibrinolytic effect [20, 21].

*S. mansoni* infection is known to induce hepatocellular injury, which in turn, leads to the release of enzymes from the injured hepatic cells into blood circulation [22]. In the present study, the significantly lower levels of AST and ALT in the liver homogenates from the infected groups may be due to the existence of the inflammatory hepatic granuloma reported to be present as a result of egg deposition and the presence of worms and their toxins. Other investigators have found increases in serum transaminases in *S. mansoni* infected animals [23]. The results obtained in this study showed that the anitinflammatory activity of berberine was slightly reflected in an improvement of the status of the bilharzial livers.

Schistosomiasis is associated with the liberation of free radicals and the disturbance of the cellular antioxidant system. It is known that antioxidant processes play an important role in mediating liver injury in schistosomiasis due to the increased production of reactive oxygen intermediates [24]. Hence, the suppressive effect of berberine on the formulation of granulomas is probably due, in part, to the fact that berberine has an antioxidant effect [25].

GSH is known to play an important role in antioxidant defence, both directly through the scavenging of reactive oxygen species, and indirectly through its function as a cofactor of antioxidant enzymes [26]. It has also been reported that schistosomiasis causes an impairment of the liver GSH content of mice, thus serving to decrease the antioxidant capacity of the liver and leading to the generation of lipid peroxides that may in turn play a central role in the pathology associated with schistosomiasis [27]. In the present study, hepatic GSH decreased significantly (by approximately 31.5%) in infected treated mice, compared to the normal control group, which indicates that schistosomiasis causes more liberation of free radicals. Berberine treatment, however, decreased both hepatic lipid peroxidation and GSH depletion strongly suggesting that berberine is an effective antioxidant in this context.

It was observed that the infection caused significant increases in the hepatic TBARS levels. Berberine treatment prevented the increase in TBARS, probably in part by scavenging the very reactive components. Moreover, high rate of oxidative processes, formation of hepatic TBARS due to the peroxidative damage to the liver microsomal membrane lipid and impairment of the antioxidant defense characterize schistosomiasis [28].

Catalase (CAT) has been regarded as a major determinant of hepatic antioxidant status by catalyzing the reduction of hydrogen peroxides and protecting tissue from highly reactive hydroxyl radicals. A decrease in CAT activity could result from inactivation by superoxide radicals and glycation of the enzymes [29]. Moreover, CAT is known to be involved in detoxification of high H_2_O_2_ concentrations, whereas glutathione peroxidase is sensitive to lower concentrations of H_2_O_2_[29].

Results obtained with berberine are a clear indication of the importance of berberine for the treatment of *S. mansoni* infection, which is comparable to that of praziquantel.

## Conclusion

Collectively, the findings of the present investigation suggest that the way in which BER exerts its beneficial effects on *S. mansoni*-induced oxidative stress may be attributed to its antioxidant activity, and that this action could find a clinical use in the treatment of hepatic dysfunction in schistosomiasis. Further studies are still necessary, however, in order to elucidate the exact mechanism of this modulatory effect, and to examine its potential therapeutic effects in more detail.

## Methods

### Animals

Swiss albino mice were bred under specified pathogen-free conditions and fed a standard diet and water *ad libitum*. The experiments were performed only with male mice at an age of 9-11 weeks; they were approved by state authorities and followed Egyptian rules for animal protection.

### Infection of mice

*S. mansoni* cercariae were from the Schistosome Biological Supply Center at Theodor Bilharz Research Institute, Imbaba, Giza, Egypt. Mice were exposed to 100 ± 10 *S. mansoni* cercariae per mouse by the tail immersion method, modified by Oliver and Stirewalt [30].

### Experimental design

Animals were allocated to six groups of eight mice each. Group one served as a control group and received water (100 μl water/mouse) by oral administration for 10 days. Group two was treated with Praziquantel (PZQ) at 500 mg/Kg via 70% glycerine on two successive days. Group three were gavaged with 100 μl of 12 mg/kg berberine chloride (one-third of the 50% lethal dose) (Sigma, St. Louis, MO, USA) [31] for 10 days. Groups four, five and six were infected with 100 ± 10 *S. mansoni* cercariae. On day 46 p.i. with *S. mansoni*, the animals of groups five and six were given PZQ and Berberine by gavage at the same doses as groups two and three, respectively. On day 55 p.i with *S. mansoni*, the animals of all groups were killed. Part of the liver was weighed and homogenized immediately to give a 50% (w/v) homogenate in an ice-cold medium containing 50 mM Tris–HCl and 300 mM sucrose, pH 7.4 [32]. The homogenate was centrifuged at 500 × g for 10 min at 4°C. The supernatant (10%) was used for the various biochemical determinations.

### Egg count in the liver

The eggs in the liver of infected mice were counted according to Pellegrino et al. [33]. In brief, the number of eggs per gram of liver tissue was determined by weighing a piece of liver (0.1 g) and divided it into four fragments, each fragment being crushed between a slide and a cover slip. The fragments were then examined by light microscope and the number of eggs counted.

### Histology of the liver

Tissue samples from the livers of mice from each of the groups were immediately fixed after animal dissection in 10% neutral buffered formalin, dehydrated and processed for paraffin sectioning. Sections were then deparaffinised and stained with hematoxylin and eosin. Histological damage was scored according to Jamshidzadeh et al. [34] as follows: 0: absent; +: mild; ++; moderate; and +++: severe. The liver activity index was estimated using a modified quantitative Ishak scoring system [17]; scores of 1-3 were assigned to cases of minimal liver damage, scores of 4-8 to mild, scores of 9-12 to moderate and scores of 13-18 to severe cases. To assess the size of tissue granuloma, the mean diameter (μm) was measured. For each group, 30 granulomas were chosen from different sections and different mice.

### Biochemical analysis

#### Aminotransferases

Colorimetric determination of alanine aminotransferase (ALT) and aspartate aminotransferase (AST) was estimated by measuring the amount of pyruvate and oxaloacetate, respectively, produced by forming 2, 4-dinitrophenylhydrazine. The colour of which was measured at 546 nm according to Reitman and Frankel [35].

#### Alkaline phosphatase

Alkaline phosphatase (ALP) was assayed in the liver homogenates using kits provided by Biodiagnostic Company (Giza, Egypt). Also, Total Bilirubin (TB) of serum was assayed according to the method of Schmidt and Eisenburg [36].

#### γ-Glutamyl transferase

γ-Glutamyl transferase (γGT) was estimated in the liver homogenates according to the method described by Szasz [37]. The substrate L-γ-glutamyl-4-nitroanilide, in the presence of glycylglycine, is converted by γGT in the sample to 4-nitroaniline which was measured at 405 nm in a Jasco-Japan-V530 spectrophotometer.

#### Glutathione

Glutathione (GSH) was determined chemically in the liver homogenates using Ellman’s reagent [38]. This method is based on the reduction of Ellman’s reagent (5,5′ dithiobis (2-nitrobenzoic acid) with GSH to produce a yellow compound. The chromogen is directly proportional to the GSH concentration and its absorbance was measured at 405 nm.

### Determination of thiobarbituric acid reactive substances

Thiobarbituric acid reactive substances (TBARS) were assayed through colorimetric tests of the liver homogenates according to the method of Ohkawa et al. [39]. In this method, TBARS was determined by using 1 ml of trichloroacetic acid 10% and 1 ml of thiobarbituric acid 0.67% which were then heated together in a boiling water bath for 30 min. TBARS were then determined by the absorbance at 535 nm and expressed as TBARS formed.

### Nitric oxide

The assay of nitric oxide (NO) in the liver homogenates was done according to the method of Berkels et al., [40]. In an acid medium, and in the presence of nitrite, the formed nitrous acid diazotises sulphanilamide, and this is then coupled with N-(1–naphthyl) ethylenediamine. The resulting azo dye has a bright reddish–purple colour which was measured at 540 nm.

### Statistical analysis

One-way ANOVA was carried out, and the statistical comparisons among the groups were performed with Duncan’s test using a statistical package program (SPSS version 17.0). All p values are two-tailed and *P* < 0.05 was considered as significant for all statistical analysis in this study.
